# Host Plant Species Mediates Impact of Neonicotinoid Exposure to Monarch Butterflies

**DOI:** 10.3390/insects12110999

**Published:** 2021-11-06

**Authors:** Cody Prouty, Paola Barriga, Andrew K. Davis, Vera Krischik, Sonia Altizer

**Affiliations:** 1 Odum School of Ecology, University of Georgia, Athens, GA 30602, USA; paobarriga@gmail.com (P.B.); akdavis@uga.edu (A.K.D.); saltizer@uga.edu (S.A.); 2 Department of Entomology, University of Minnesota, St. Paul, MN 55108, USA; krisc001@umn.edu

**Keywords:** clothianidin, imidacloprid, *Danaus plexippus*, sub-lethal effects, toxicity, *Asclepias*, milkweed, flight

## Abstract

**Simple Summary:**

Neonicotinoids are the most widely used insecticides in North America and many studies document the negative effects of neonicotinoids on bees. Monarch butterflies are famous for their long-distance migrations, and for their ability to sequester toxins from their milkweed host plants. The neonicotinoids imidacloprid and clothianidin were suggested to correlate with declines in North American monarchs. We examined how monarch development, survival, and flight were affected by exposure to neonicotinoids, and how these effects depend on milkweed host plant species that differ in their cardenolide toxins. Monarch survival and flight were unaffected by low and intermediate neonicotinoid doses. At the highest dose, neonicotinoids negatively affected monarch pupation and survival, for caterpillars that fed on the least toxic milkweed. Monarchs fed milkweed of intermediate toxicity experienced moderate negative effects of high insecticide doses. Monarchs fed the most toxic milkweed species had no negative consequences associated with neonicotinoid treatment. Our work shows that monarchs tolerate low neonicotinoid doses, but experience detrimental effects at higher doses, depending on milkweed species. To our knowledge, this is the first study to show that host plant species potentially reduce the residue of neonicotinoid insecticides on the leaf surface, and this phenomenon warrants further investigation.

**Abstract:**

Neonicotinoids are the most widely used insecticides in North America. Numerous studies document the negative effects of neonicotinoids on bees, and it remains crucial to demonstrate if neonicotinoids affect other non-target insects, such as butterflies. Here we examine how two neonicotinoids (imidacloprid and clothianidin) affect the development, survival, and flight of monarch butterflies, and how these chemicals interact with the monarch’s milkweed host plant. We first fed caterpillars field-relevant low doses (0.075 and 0.225 ng/g) of neonicotinoids applied to milkweed leaves (*Asclepias incarnata*), and found no significant reductions in larval development rate, pre-adult survival, or adult flight performance. We next fed larvae higher neonicotinoid doses (4–70 ng/g) and reared them on milkweed species known to produce low, moderate, or high levels of secondary toxins (cardenolides). Monarchs exposed to the highest dose of clothianidin (51–70 ng/g) experienced pupal deformity, low survival to eclosion, smaller body size, and weaker adult grip strength. This effect was most evident for monarchs reared on the lowest cardenolide milkweed (*A. incarnata*), whereas monarchs reared on the high-cardenolide *A. curassavica* showed no significant reductions in any variable measured. Our results indicate that monarchs are tolerant to low doses of neonicotinoid, and that negative impacts of neonicotinoids depend on host plant type. Plant toxins may confer protective effects or leaf physical properties may affect chemical retention. Although neonicotinoid residues are ubiquitous on milkweeds in agricultural and ornamental settings, commonly encountered doses below 50 ng/g are unlikely to cause substantial declines in monarch survival or migratory performance.

## 1. Introduction

Neonicotinoids are a class of synthetic neuroactive insecticides similar in structure to nicotine; they have come into widespread use since the late 1990s and are presently the most widely used class of insecticide in the world [1]. Neonicotinoids such as clothianidin, imidacloprid, and thiamethoxam are widely used in row crops in North America, as well as on orchards, vegetables, and ornamental plants, and can be applied through seed treatment, soil drenching, and foliar application [2]. The compounds persist in the environment for many months, with half-lives from hundreds to thousands of days in the absence of UV exposure [3]. In the presence of UV light, neonicotinoids degrade quickly and this photodegradation is dependent on water constituents [4]. They can be incorporated into plant tissue through uptake in the roots and expressed persistently in leaves and flowers [5]. Seed and soil treatments are commonly employed for application of neonicotinoids; only about 5% of the active ingredient ends up in the target plant, the rest enters the environment [6].

Neonicotinoids bind to nicotinic acetylcholine receptors in insects, causing paralysis and death [7], and are highly effective against many sucking, leaf chewing, and soil insects [8]. These insecticides have many sublethal effects that alter movement, behavior, and navigation [9]. Owing to strong negative effects on honeybees and bumblebees exposed through pollen and nectar (and sub-lethal effects detected as low as 2–20 ng/mL; [10,11,12,13,14]), neonicotinoids are now banned in the European Union.

Monarchs (*Danaus plexippus*) are charismatic and iconic insects, with scientific interest in their biology stemming from their long-distance yearly migrations and their ability to sequester cardenolide toxins produced by their milkweed host plants, e.g., [15,16,17]. Declines of winter colonies of North American monarchs have caused concern for the persistence of their migration [18,19]. While some evidence has suggested these declines stem from issues related to habitat loss during summer [20,21,22], other research points to problems faced during the fall migration [23,24,25,26]. In particular, neonicotinoids have been suggested as a potential cause of decreased monarch migration success [27,28,29]. Some studies cited the increasing use of neonicotinoids as a correlational factor with declines in eastern monarchs [28,30], and another found that western monarch declines were greater where pesticide use and habitat loss were higher [31].

Monarchs can be exposed to neonicotinoids in agricultural environments through drift from foliar applications or soil leaching from seed treatments at planting [32]. Neonicotinoid residues are reported in field surveys of wildflowers and milkweeds [33,34,35,36] and are found up to 48 ng/g on nectar plants and up to 56.5 ng/g on milkweed plants in field margins. Monarch caterpillars often feed on milkweed in agricultural fields and can be exposed to insecticide and herbicide foliar spraying [37]. Chronic studies to date testing effects of neonicotinoids on monarchs have generally found moderate [38,39] or low toxicity of several neonicotinoids [40], with the exception of one study, which estimated high clothianidin toxicity [41]. A complicating factor is that these studies differed in the insecticides used, application methods, exposure stage, milkweed species used as host plants, and response variables recorded (summarized in Table S1). Further work is needed to resolve the differences reported in studies to date, particularly in reference to effects of different host plant species and methods of exposure [42,43,44].

Certain insects have evolved detoxification systems for coevolved plant compounds [45]. Monarchs in particular can tolerate and sequester cardenolides produced by milkweeds into their bodies to deter predation [46]. This comes at a cost, as high cardenolide doses can reduce caterpillar survival and development [47]. The pathway that monarchs and other species that feed on milkweed use to deal with cardenolides has been studied [47,48], though to date there has been no research evaluating how cardenolide intake influences the physiological impacts of chemical insecticides. For example, neonicotinoids and cardenolide toxins could potentially interact in ways that amplify the effects of insecticides or dampen their overall effects, if the pathways used to tolerate cardenolides also respond to neonicotinoids. If so, and since milkweed species have differing levels of cardenolides [49,50], the impact of neonicotinoids on monarchs may vary with milkweed type. Moreover, varying leaf chemistry and physical properties among milkweed species could affect cardenolide retention times and exposure levels.

Here we examined how neonicotinoid consumption by caterpillars influences monarch development and survival, and whether insecticide impacts vary among commonly encountered milkweed species. We first exposed monarchs reared on less toxic swamp milkweed to field-relevant doses (0.075 and 0.225 ng/g, estimated) of clothianidin and imidacloprid. After finding no effect of exposure to these low doses on monarch development, survival, or flight performance, we conducted a second experiment with higher neonicotinoid doses (4–70 ng/g, Table 1) applied across three milkweed species representing low (swamp milkweed, *A. incarnata*), moderate (common milkweed, *A. syriaca*), and high (tropical milkweed, *A. curassavica*) average cardenolide content. Differences in cardenolide concentrations between these milkweed species are well-established in the literature, with swamp milkweed having consistently low cardenolides and tropical having consistently high. Common milkweed shows more variability, but consistently falls between this range [51,52,53,54]. We again examined monarch development and survival to the adult stage and tested grip strength [55] as an indicator of physical performance. Using the same concentrations from Experiment 2, we conducted a small bioassay on bumblebees to confirm the toxicity of clothianidin and imidacloprid used in our monarch studies (Supplemental Materials).

Based partly on findings by Krishnan et al. [40,56], we predicted that monarchs would be more tolerant of neonicotinoids (e.g., the lethal dose would be higher) than previous studies indicated, such as Pecenka and Lundgren [41]. In terms of flight performance, we predicted that monarchs exposed to neonicotinoids as larvae would fly shorter distances and fly slower, perhaps because of impaired neurological functions. We also considered that even if monarchs do not retain the toxins from neonicotinoids into adulthood, it is possible that, if there are effects on development, then exposed monarchs could still show reduced flight performance. Finally, we predicted that milkweeds with different cardenolide concentrations would influence monarch tolerance of neonicotinoids, with more toxic plant species either enhancing monarchs’ ability to tolerate other toxins or intensifying the negative neonicotinoid effects.

## 2. Materials and Methods

### 2.1. Monarchs and Host Plants

We used captive-reared monarchs that were non-inbred F3 descendants of wild-caught fall migrants from Athens, GA, and St. Marks, FL, USA in October 2017 (Experiment 1) and October 2018 (Experiment 2). Adult monarchs were mated in 0.6 m^3^ mesh cages and fed ad libitum with a 20% honey-water solution. Mated females oviposited onto *A. incarnata* cuttings, and larvae remained on natal stalks until second instar. We obtained 3–4 outcrossed genetic lineages of monarchs per experiment.

Milkweed plants were raised from seeds obtained from Prairie Moon nursery (swamp, *Asclepias incarnata* and common, *A. syriaca*) and the vendor SEEDS2GO (tropical, *A. curassavica*) and planted into 12.5 cm diameter pots. Milkweed species used here are common throughout much of the eastern migratory monarchs’ breeding range, although tropical milkweed is largely planted in gardens and can serve as an ecological trap to monarchs based on past work [57]. Plants were pruned several times prior to each experiment and received bi-monthly pelleted fertilizer and weekly spraying with insecticidal soap to control aphids and thrips. Greenhouse temperatures fluctuated between 15 °C and 35 °C, with a 16:8 light:dark cycle under broad spectrum lights.

### 2.2. Neonicotinoid Doses

One milligram each of clothianidin and imidacloprid (MilliporeSigma, St. Louis, MO, USA) was dissolved separately into 0.5 L of distilled water to achieve a 2 ppm (mg/L) stock solution, which was further diluted to doses of 5, 15, 50 and 500 ng/mL using distilled water. We measured out multiple dilutions throughout each experiment from a single stock solution mixed at the start of each experiment. Stock solutions were held at 4 °C for up to 12 days per experiment, and original (solid) chemicals were held at 22 °C for up to 18 months. For dose verification, solutions and leaf samples were sent frozen to the USDA Agricultural Marketing Service, Science and Technology Laboratory Approval and Testing Division’s National Science Laboratories, Gastonia, NC. The samples were extracted using a refined methodology for the determination of neonicotinoids using an approach of the official pesticide extraction method (AOAC OMA 2007.01), also known as the QuEChERS method, and analyzed by liquid chromatography coupled with tandem mass spectrometry detection (LC/MS/MS). Quantification was performed using external calibration standards prepared from certified standard reference material. The instrumentation used was an Agilent LC/MS/MS (1200 pumping systems and models 6420 or 6430 detection units). Each 3 g subsample of a homogenized sample was fortified with one or more process control standard (PCS). The analytes of interest and PCS(s) were extracted from the samples by high-speed grinding in an acidified acetonitrile and water mixture followed by a “clean-up” to remove some matrix components and filtration to remove particulates. Enhance matrix reduction (Agilent EMR) material was utilized to remove lipids from applicable matrices. Separate aliquots of extract were analyzed for pesticide residue by gas chromatography (GC) and liquid chromatography (LC) techniques utilizing mass selective detection systems.

### 2.3. Experiment 1: Effects of Low-Dose Larval Exposure on Monarch Development and Flight

To test responses to low doses of clothianidin and imidacloprid, caterpillars were raised singly on greenhouse-raised *A. incarnata*. Five treatments (N = 227 larvae) included a distilled water control (35 larvae), clothianidin 5 and 15 ng/mL (48 larvae/treatment), imidacloprid 5 and 15 ng/mL (48 larvae/treatment) applied directly to leaves. Larvae remained on natal milkweed stalks until they reached mid-second instar and were then transferred to 0.5 L plastic containers with mesh screen lids. Each day for 5 days we painted milkweed cuttings (removing all but four leaves per container) with 15 µL insecticide solution per leaf and fed them to larvae. Solution was administered using a micro-pipettor and spread across the leaf surface with a small craft paintbrush, resulting in estimated concentrations of 0.075 and 0.225 ng/g for the low and high concentrations, respectively. If monarchs consumed the treated cutting, they were fed ad libitum with untreated milkweed until the next day. Tools used for feeding and applying insecticides were physically separated for each treatment and were exposed to UV light daily for 2 h to degrade residual neonicotinoids.

After 5 consecutive days of treatment application, late-instar monarchs were fed *A. incarnata* clippings ad libitum until pupation (3–4 additional days). Containers were checked twice daily for deaths or pupation. Monarch pupae were weighed 5 days post-pupation to the nearest 0.001 g using an analytical balance. We recorded eclosion date and sex, checked for infection by the protozoan *Ophryocystis elektroscirrha*, and held adult monarchs in individual glassine envelopes at 24 °C until the day of flight testing (below). We fed monarchs by hand a 20% honey-water solution each day for 5 days after eclosion.

We measured monarch flight indoors during May–June 2018 using a tethered flight mill (see Figure S1 for schematic) in a 9 m^2^ room at 29.7 °C (range 27.8–31.4 °C) between 1000 and 1730 h. Five to six days post-eclosion, we glued lightweight steel wires (15 lb test) to the dorsal side of each monarchs’ thorax using rubber cement, following Bradley and Altizer [58]. As per Schroeder et al. [59], the average mass of the wire attachment was 0.19 g (range 0.10–0.33 g). Monarchs were placed into 0.6 m^3^ mesh cages to adjust to the weight of the wire, with 20% honey-water provided ad libitum). The flight mill was constructed as described in Bradley and Altizer [58] and Fritzsche McKay, Ezenwa [60] from a 120 cm lightweight carbon rod with a diameter of 3 mm (4.23 m circumference) attached to a frictionless steel pivot (Figure S1). We tethered monarchs to one end of the horizontal rod, and a flag at the opposite end passed through an infrared beam on a photo-gate to estimate flight velocity per revolution (m/s; software PASCO Capstone). Windows were covered with white paper to limit sun angle cues during flight, and we positioned floor lamps to provide an even distribution of light.

Monarchs were flown on the apparatus for a maximum of 1 h. Monarchs that stopped flying for more than 5 s were agitated with a gust of air. If the monarch did not resume flight after three agitations, the flight was terminated. For each flight, we calculated total distance (km) flown based on the number of revolutions. We calculated average flight velocity (km/h) by dividing the total distance by time in flight. Next, we scanned each live monarch on a standard flatbed scanner for quantification of wing dimensions. We measured wing area (in mm^2^) using Fovea Pro 4.0 plugins for Photoshop CS2 from the scanned images of the adults [61].

### 2.4. Experiment 1 Analyses

Analyses were performed in R version 3.5.3. We first used general linear mixed models to test for relationships between neonicotinoid exposure and monarch development. Neonicotinoid type was categorized as a 5-level factorial variable (levels = control, 5 and 15 ng/mL imidacloprid, 5 and 15 ng/mL clothianidin). We analyzed pupal weight, larval growth rate (pupal mass/days to pupation), wing area, and adult monarch weight as response variables, using GLMMs with normal error structures and the following model structures in the lme4 package [62]: [response variable = insecticide group + genetic lineage (random effect)]. Sex was included as a separate predictor for adult response variables. For flight variables, we analyzed distance flown, average speed, time spent flying, and power (from the calculation described in Fritzsche McKay, Ezenwa [60]). Flight distance and time were log-transformed to normalize the error variance. Models for flight variables included the following covariates: adult age on date flown, wire weight, weight before flight, and wing loading—together with insecticide group (5 levels), sex, and genetic lineage (random effect).

### 2.5. Experiment 2: Effects of High-Dose Larval Exposure on Monarch Development and Grip Strength

To test the extent to which neonicotinoid effects on monarch survival and development depend on host plant species, we reared monarchs on potted milkweeds inside a greenhouse, using swamp milkweed (low cardenolide levels, 0.07 µg/mg), common milkweed (moderate cardenolide levels, 0.3–0.6 µg/mg), and tropical milkweed (high cardenolide levels, 2.1 µg/mg; [63]). Differences in cardenolide concentrations for the milkweed species used in this study were consistently established by prior work; as such, we did not measure them directly here. Potted plants exposed to natural sunlight were used to increase the relevance of findings to conditions monarchs experience in nature and to minimize the handling of monarchs once they were placed on plants. We again used clothianidin and imidacloprid, with five exposure treatments (N = 240): control (water only), clothianidin 50 and 500 ng/mL, and imidacloprid 50 and 500 ng/mL for both swamp (24 monarchs per treatment) and tropical milkweed (15 monarchs per treatment). For common milkweed (15 monarchs per treatment) only two treatments were used: control and 500 ng/mL (Table S2).

We used a pump sprayer to administer 60–70 mL/plant of clothianidin and imidacloprid solutions (distilled water for controls) on the tops and bottoms of leaves of pruned 0.6 m tall plants. Second instar monarch larvae (from 5 outcrossed lineages) were reared individually on these potted milkweeds after plants were sprayed and dried. For this experiment, larvae were not reared in containers but directly on the potted plants; the potted plants, including larvae, were enclosed in a clear acrylic tube (0.5 mm thick, 1 m tall, 12–13 cm in diameter; Figure S2) with mesh fabric fastened to the top. Pots were placed into solid-bottom trays to retain water and hydrate plants. Trays were randomly organized across four greenhouse benches in two adjacent rooms. Owing to the known decay of neonicotinoids in UV light, plants were re-sprayed with 20 mL fresh solution per plant 5 days after initial treatment. This reapplication was intended to reestablish doses similar to day 0 concentrations. Monarchs remained on plants during the second spray, but we avoided the direct spraying of caterpillars. We acknowledge that some monarchs likely had cuticular exposure before the solution evaporated but this was not quantified.

Monarchs were observed daily to record survival and pupation. After 5 days post-pupation, pupae were weighed to the nearest 0.001 g using an analytical balance. We recorded pupal deformity or discoloration on a 0–3 scale (0 = normal pupal color and shape; 1 = mild discoloration or deformity; 2 = moderate discoloration or deformity; 3 = failure to complete ecdysis; Figure S3). We recorded monarch eclosion date and sex and checked all monarchs for infection by the protozoan *O. elektroscirrha*. As in Experiment 1, we scanned adult monarchs and measured wing area using digital image analysis.

Monarch grip strength was measured following Davis et al. [55] using a device that detects how much force in newtons (N) is exerted when monarchs pull on a rod attached to a force gauge. Briefly, an observer holds a monarch by the closed wings and lowers it to the rod until it grips with all four tarsi. The observer then gently pulls the monarch directly upwards until it releases from the rod. This was repeated five times per individual, to obtain an average measure of releasing force (i.e., grip strength). Strength trials were performed blind (we reassigned monarch identification numbers) to limit observer bias.

### 2.6. Experiment 2 Analysis

We analyzed outcomes of control, 4–7 ng/g and 33–70 ng/g applications across all three host plant species, coding neonicotinoid treatment as a 5-level fixed factor (control, 5–7 ng/g imidacloprid, and 4–5 ng/g clothianidin 33–47 ng/g imidacloprid, and 51–70 ng/g clothianidin). We tested the following response variables: larval growth rate (pupal mass/days to pupation), forewing area, grip strength, and pupal deformity (0–3 scale); using the following model: response variable = insecticide + milkweed species + insecticide×milkweed + block (5 greenhouses) + monarch lineage (random effect). Analyses based on adult data (forewing area and grip strength) included sex as a main effect. We analyzed the proportion of monarchs that eclosed (0/1) using a GLM with binomial distributions for the error structure. In any instance with an insignificant block effect, the variable was removed. Multiple comparisons used Satterthwaite’s method for t tests.

## 3. Results

### 3.1. Realized Neonicotinoid Doses

We first report the realized neonicotinoid doses fed to monarchs based on analysis of samples sent to the USDA lab. For Experiment 1, leaves were painted and collected 1 hour later; residue assays showed that only trace amounts (<3 ng/g of leaf) of the intended 5 and 15 ng/mL clothianidin and imidacloprid were detected on milkweed leaves (Table 1). For Experiment 2, leaves were sprayed and collected 1 hour later on day 0 and were again collected at 4 days (before second spraying). Residue assays showed that leaves had roughly 10% of the applied dose of clothianidin and imidacloprid on the day plants were treated, with a range of 4–51 ng/g for swamp milkweed and 4–70 ng/g for tropical milkweed. On day 4 post-application, swamp milkweed had a range of 4–31 ng/g on leaves and tropical milkweed had trace amounts detected (Table 1). Neonicotinoids were not detected on control leaves. Thus, the detected doses were lower than the applied dose in both experiments. Importantly, the detected doses are ecologically relevant, given that field studies found up to 56.5 ng/g of clothianidin in wild milkweed leaves on field margins [34,43].

### 3.2. Experiment 1: Effects of Low-Dose Larval Exposure on Monarch Development and Flight

**Figure 1:**
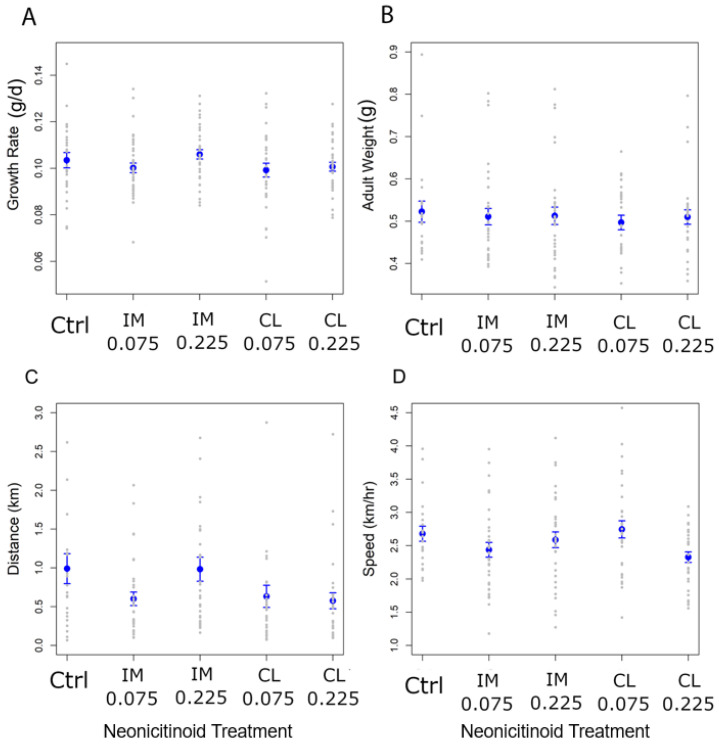
Experiment 1. Monarch response variables shown for each neonicotinoid chemical and dose reared on swamp milkweed. IM = imidacloprid and CL = clothianidin. Numbers under the neonicotinoid treatment indicate the estimated concentration on leaf tissues in ng/g, though realized dose for each treatment was below detection levels (Table 1). Gray dots represent each individual monarch; error bars represent standard errors. (**A**) Larval growth rate (g/d); (**B**) Weight (g) of adult monarchs upon eclosion; (**C**) Flight distance in km (flights were terminated after a maximum of 1 h); (**D**) Flight speed, measured as total distance in km over flight duration in hours.

A total of 165 (72.7%) of the 227 monarchs placed on plants at second instar survived to eclosion when reared on swamp milkweed at low doses of 5 ng/g (detected dose <3 ng/g) and 15 ng/g (detected dose <3 ng/g). No larval or adult response variables differed significantly among the insecticide treatments (Table 2; Figure 1A,B). We selected 139 of the 165 adult monarchs to measure flight (20 controls, and 21–31 per insecticide treatment; data were excluded from 2 control and 14 insecticide-treated monarchs that did not fly for a minimum of 3 min). Monarchs across all treatments flew an average distance of 0.745 km ± 0.06 SE, at a speed of 2.55 km/h ± 0.05 SE, and for a duration of 16.92 min ± 1.25 SE. Flight measures were not significantly different across the treatments (Table 2; Figure 1C,D).

### 3.3. Experiment 2: Effects of High-Dose Larval Exposure on Monarch Development and Grip Strength

Of the 240 monarchs placed onto plants in Experiment 2, 210 (87%) survived to pupation, 208 (86.67%) eclosed as adults, and 206 (86%) had wings scanned and participated in the grip strength test. Larval growth rate (g/d) was significantly lower for monarchs reared on common milkweed and was faster for monarchs that fed on tropical and swamp milkweed, and larval growth-rate did not differ according to insecticide treatment (Table 3).

**Figure 2:**
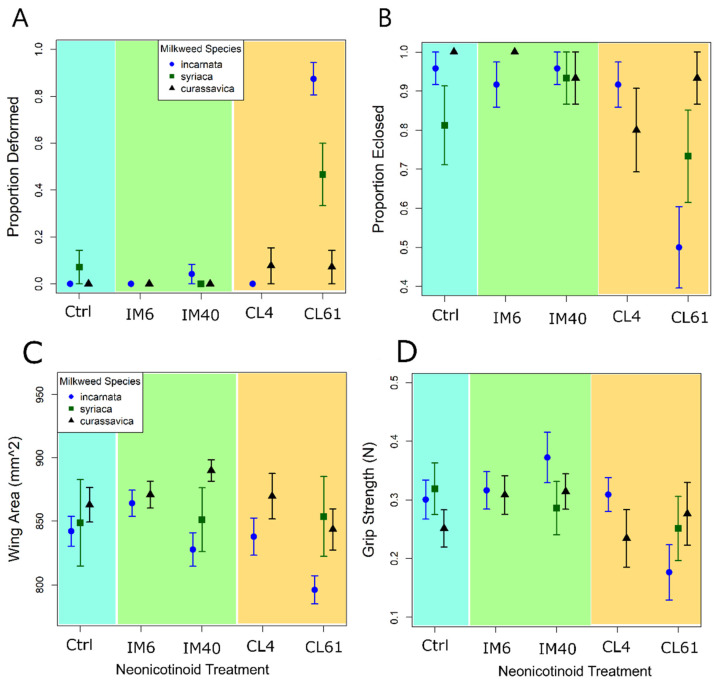
Experiment 2. Effects of detected dose of neonicotinoids and milkweed species on monarch deformity, eclosion, grip strength, and wing area. Numbers under the neonicotinoid treatment indicate the realized dose (ng/g) on leaf tissues. IM = Imidacloprid, CL = Clothianidin. Swamp milkweed (*A. incarnata*) is in blue circles, tropical (*A. curassavica*) is in black triangles, and common milkweed (*A. syriaca*) is in green squares. (**A**) The proportion of monarchs that showed deformed pupae; (**B**) The proportion of monarchs that eclosed successfully; (**C**) Forewing area (mm^2^) of monarchs; (**D**) Grip strength (newtons) as measured by monarchs’ ability to pull a rod.

Nearly all monarchs that fed on swamp milkweed (*A. incarnata*, low cardenolides) and were exposed to the highest dose of clothianidin (500 ng/g, applied dose; 51 ng/g, detected dose) experienced problems during pupation (Figure 2B), with many failing to shed their larval integuments (failed ecdysis; Figure S3D). Nearly 50% of all monarchs that fed on common milkweed (*A. syriaca*, intermediate cardenolides) and were exposed to the highest dose of clothianidin also experienced problems during pupation or showed pupal discoloration and deformity (Figure 2A). In contrast, monarchs reared on tropical milkweed (*A. curassavica*, high cardenolides) pupated normally, irrespective of insecticide treatment (Figure 2A). The interactive effects of milkweed species and insecticide treatment on pupal deformity were highly significant (Table 3). However, those treated with imidacloprid and any concentration (5–47 ng/g) showed little to no negative response.

In the high-dose (500 ng/g applied dose; 33–47 ng/g imidacloprid; 51–70 ng/g clothianidin) neonicotinoid treatments, over 90% of monarchs that fed on tropical milkweed survived to the adult stage, compared to 70 and 48% of monarchs that fed on common and swamp milkweed, respectively. The interaction between host plant and insecticide treatment on adult survival was significant (Figure 2B, Table 3). Adult wing area was lowest for monarchs reared on swamp milkweed (Figure 2C, Table 3). Larvae exposed to the highest dose of clothianidin (51 ng/g) and fed swamp milkweed had significantly smaller wing area than control monarchs (Figure 2C; t(204) = −2.607, *p*-value < 0.01). The main effect of insecticide treatment on wing area was significant, but not the interaction between insecticide treatment and milkweed species (Table 3).

The grip strength of adults was lowest for monarchs reared on swamp milkweed and treated with 51 ng/g of clothianidin (Figure 2D; t(187) = −2.634, *p* = 0.009). However, analysis showed no significant main or interactive effects of insecticide treatment on grip strength (Table 2). Males showed significantly greater grip strength than females (Table 3).

## 4. Discussion

The impact of neonicotinoid insecticides on monarch butterflies, both in their breeding range and during the long-distance fall migration, has been identified as a potential conservation concern [9,27,28,30]. Data from this study indicated that field residues below 70 ng/g imidacloprid and 51 ng/g clothianidin should not impact monarch development, grip strength, or wing size. Based on the results of Experiment 1, neonicotinoid solutions of 5 and 15 ng/mL applied to milkweed leaves resulted in residues below the limits of detection on the day of treatment. Monarch caterpillars ingesting these trace amounts experienced no lethal or sub-lethal effects on development, adult size, or flight ability. In Experiment 2, at the highest doses of 51–70 ng/g (a concentration comparable to the upper end of levels in the agricultural and nursery industries), negative effects of clothianidin began with the onset of pupation and depended strongly on host plant species and insecticide type and dose. Negative effects on development and size were strongest for monarchs that fed on swamp milkweed treated with high-dose clothianidin. In contrast, caterpillars that fed on the highest cardenolide milkweed (tropical, *A. curassavica*) and those treated with imidacloprid and any concentration (5–47 ng/g) showed little to no negative response to neonicotinoid exposure. The same solutions used in Experiment 2 dramatically reduced bumble bee survival within a period of 4 hours (Supplemental Materials).

In Experiment 2, neonicotinoid residue on swamp milkweed was maintained at 10% of the application dose at least 4 days after treatment. At the 500 ng/mL application of clothianidin (51 ng/g detected dose), nearly all caterpillars showed signs of pupal deformity, including failure to shed larval integuments, and only half of the monarchs in this treatment eclosed as adults. Similar rates of pupal deformity and ecdysis failure following exposure to high clothianidin doses were reported by another recent study [40]. On tropical milkweed, residue was reduced to trace amounts by day 4 post-application, and monarchs feeding on this high-cardenolide host plant showed normal pupation and over 90% eclosion success. Monarchs that fed on common milkweed (*A. syriaca*, intermediate cardenolides) showed intermediate rates of pupal deformity (50%) and eclosion success (70%). In contrast, caterpillars that fed on milkweed treated with imidacloprid (5–47 ng/g), showed no negative response.

To our knowledge, this is the first study to show that host plant species mediates the impact of neonicotinoid insecticides. The mechanism behind this effect could be physiological in nature and perhaps involve the natural cardenolide tolerance possessed by monarchs. Alternatively, physical or chemical properties of the leaves of the different milkweed species could affect how well and for how long the pesticides remain active, thereby affecting how much is consumed by larvae. Tropical milkweed, for example, has smooth, waxy leaves, and chemicals applied by spraying may not be as readily absorbed. Additionally, it has been shown that neonicotinoids, particularly clothianidin, applied to plants can alter their gene expression for defensive chemicals [64], resulting in changes in plant chemistry. Given that different species of milkweed produce more or less cardenolides, it is possible that their gene expression could be differentially affected by clothianidin. In the current study, we preserved insufficient leaf biomass to comprehensively analyze chemical residues for both neonicotinoids and cardenolides across the separate milkweed species. Importantly, three previous studies [38,39,56] showed similar neonicotinoid effects using different milkweed species, although these studies did not compare multiple host plant species within the same experiment.

Although, monarchs reared on tropical milkweed showed minimal negative effects of neonicotinoid exposure, this should not be taken as an argument to increase the use of this plant in backyard gardens or to purchase it to protect monarchs from harm from these chemicals. Tropical milkweed has been shown to be an ecological trap for migrating monarchs, leading to increases in infection by a debilitating protozoan, reducing the induction of reproductive diapause prior to fall migration, and reducing wing elongation [65,66,67,68]. Thus, we argue that the negative effects of tropical milkweed for monarch health and migration vastly outweigh the potential benefits regarding neonicotinoid tolerance.

The literature surrounding neonicotinoids and monarchs is growing quickly, though differences in methodologies and treatments among studies make it difficult to compare outcomes. Indeed, past research examining lethal doses of neonicotinoids varied between feeding intact stems, leaf discs, and treating entire plants with neonicotinoids. In our study, monarchs fed on whole plants and intact stems showed no negative effects on survival up to 33 ng/g imidacloprid and 51 ng/g clothianidin reared on *A. incarnata*. Krischik et al. [42] showed significantly reduced survival of 90% by 7 days in monarch larvae fed 6000 ng/g imidacloprid in *A. curassavica* leaves. Knight et al. [69] detected 3% reduced survival of monarch larvae in a single field of milkweed growing near seed-treated corn that resulted in 14 ng/g clothianidin (range 7–21 ng/g *A. syriaca*). Meanwhile, Bargar et al. [38] found no effects on larvae fed *A. incarnata* plants treated with a soil application that resulted in around 66–205 ng/g clothianidin. Olaya-Arenas et al. [39] found no effect of clothianidin on survival or developmental time below 54 ng/g leaf on *A. syriaca*. In leaf disc studies, Pecenka and Lundgren [41] found an LC50 of approximately 9.8 ng/g (converted in Krishnan et al. [56]) for clothianidin on *A. incarnata*. Krishnan et al. [40] found an acute LC50 of 9.4 ug/g for imidacloprid and 0.8 ug/g for clothianidin on *A. curassavica*, which was similar to Krischik et al.’s [42] finding of 6 ug/g imidacloprid on *A. curassavica*. See Krishnan et al. [56] for further details and summaries of findings from acute and chronic exposure to neonicotinoids.

In this study, the absence of negative effects of neonicotinoids on monarch development and survival (except for the highest doses of clothianidin, which was at the upper limit of residues found on field-collected plants) indicated that exposure to this class of insecticides likely does not dramatically reduce the breeding population of monarchs [38]. Furthermore, our results concerning monarch flight ability also indicated that neonicotinoids might be less relevant to monarch migration than previously suspected, e.g., [40,69]. This conclusion was in agreement with recent findings from field-tracked monarchs that had been exposed to neonicotinoids [29]. Therefore, it is becoming clear that neonicotinoid exposure at concentrations typically found in milkweed does not hinder monarch flight and by extension, migration success.

There are a variety of contexts under which monarchs and other pollinators could become exposed to higher doses than those found at field margins (and what was used here). Cowles and Eitzer [5] found that milkweed treated with products usually bought by gardeners contains neonicotinoids that can lead to concentrations of up to 1000 ng/mL in the plants’ nectar. If gardeners use these products to deter pest insects, monarchs and other pollinators could be exposed to high doses via this route (i.e., in the nectar). Given the clear reductions in bee mobility following insecticide exposure, e.g., [70,71], and the known presence of neonicotinoids along agricultural field margins, e.g., [6,36,72], it seems plausible that longer-term pesticide exposure at both larval and adult stages could still have impacts that would be felt during fall migration, such as reduced adult longevity (i.e., monarchs would not live long enough to reach their destination). This would be consistent with lab-based findings elsewhere [73].

## 5. Conclusions

Finally, our findings pointed to several areas for further investigation. First, neonicotinoids are not the only insecticides that migrating adults or feeding larvae encounter. Field surveys showed numerous insecticides, herbicides, and fungicides on wild flowers and/or milkweeds [33] that can act additively and/or synergistically and increase toxicity. Second, it is important to ask whether neonicotinoid exposure could amplify the negative fitness consequences of other environmental stressors, such as food limitation, thermal stress, or parasite infection. For example, infection by the protozoan *O. elektroscirrha* is known to significantly reduce monarch survival, body size, and flight performance [58], which can lead to higher mortality of monarchs during migration [74]. It is possible that neonicotinoids could intensify the negative effects of infection on monarch flight speed and duration. It is also important to consider that our study reared monarchs under low density, with ample food and ideal temperatures during development. An experiment that compares these effects in the field, under cases of food limitation or other sub-optimal conditions, would be important in addressing whether these effects hold up across a range of environmental circumstances.

## Figures and Tables

**Table 1:** Residue of neonicotinoids detected in leaves (Experiments 1 and 2) and solutions (Experiment 2) relative to applied dose. Leaves were stored and shipped frozen for residue analysis to the USDA, AMS lab, Gastonia, NC. IM = Imidacloprid, CL = Clothianidin, ND = not detected, NA = not applicable. Limits of detection were: 3 ng/g CL and 2 ng/g IM.

Expt	Sample	Neonic	Applied Dose (ng/mL)	Detected Dose (ng/g)	Milkweed Species	Collection Day
1	leaf	IM	5	trace	swamp	0
1	leaf	IM	15	trace	swamp	0
1	leaf	CL	5	ND	swamp	0
1	leaf	CL	15	trace	swamp	0
2	leaf	CL	500	51	swamp	0
2	leaf	CL	50	4	swamp	0
2	leaf	IM	50	7	swamp	0
2	leaf	IM	500	33	swamp	0
2	leaf	Control	0	ND	swamp	0
2	leaf	Control	0	ND	tropical	0
2	leaf	IM	50	5	tropical	0
2	leaf	IM	500	47	tropical	0
2	leaf	CL	50	4	tropical	0
2	leaf	CL	500	70	tropical	0
2	leaf	IM	500	25	swamp	4
2	leaf	IM	50	5	swamp	4
2	leaf	CL	500	31	swamp	4
2	leaf	CL	50	4	swamp	4
2	leaf	IM	50	trace	tropical	4
2	leaf	IM	500	trace	tropical	4
2	leaf	CL	500	trace	tropical	4
2	leaf	CL	50	trace	tropical	4
2	solution	CL	50	36	NA	NA
2	solution	IM	50	28	NA	NA
2	solution	CL	500	531	NA	NA
2	solution	IM	500	386	NA	NA

**Table 2:** Results of general linear models investigating predictors of monarch development and flight in Experiment 1. Monarch lineage was included as a random effect in all models. Neonicotinoid treatment was included as a fixed factor with 5 levels as described in the Methods section. Significant variables appear in bold.

Response Variable	Predictors	Mean Sq	DF	F Value	*p*-Value
Pupal mass	Neonicotinoid treatment	3.56 × 10^−2^	4	1.59	0.180
	**Sex**	**1.99 × 10^−1^**	**2**	**8.93**	**<0.001**
Larval growth rate	Neonicotinoid treatment	3.21 × 10^−4^	4	2.05	0.089
	**Sex**	**1.06 × 10^−3^**	**2**	**6.8**	**0.002**
Adult mass	Neonicotinoid treatment	2.30 × 10^−3^	4	0.21	0.934
	Sex	1.42 × 10^−7^	1	0.00	0.991
Distance flown	Neonicotinoid treatment	6.01 × 10^−1^	4	1.08	0.372
	**Sex**	**2.40**	**1**	**4.31**	**0.040**
	Pre-flight weight	2.1 × 10^−4^	1	0.00	0.984
	Wire weight	1.91 × 10^−1^	1	0.34	0.560
	Age at flight (days)	1.90 × 10^−2^	1	0.03	0.854
Flight duration	Neonicotinoid treatment	6.48 × 10^−1^	4	1.40	0.240
	**Sex**	**1.83**	**1**	**3.93**	**0.0498**
	Pre-flight weight	5.09 × 10^−2^	1	0.11	0.741
	Weight of wire	2.53 × 10^−1^	1	0.54	0.462
	Age at flight (days)	7.01 × 10^−2^	1	0.15	0.699
Flight speed	Neonicotinoid treatment	4.84 × 10^−2^	4	1.74	0.145
	Sex	1.88 × 10^−2^	1	0.68	0.413
	Pre-flight weight	6.19 × 10^−2^	1	2.23	0.138
	Weight of wire	3.66 × 10^−4^	1	0.01	0.909
	Age at flight (days)	1.03 × 10^−1^	1	3.73	0.056

**Table 3:** Results of general linear models investigating predictors of monarch growth, survival (eclosed), and deformity in Experiment 2, for all 3 milkweed species (swamp, common, and tropical) and 5 insecticide treatments (clothianidin and imidacloprid) (Table S2). Monarch lineage was included as a random effect in all models. Neonicotinoid treatment was included as a fixed factor with 3 levels as described in the Methods section. Survival (eclosed) was treated as a binomial variable (binomial errors, logit link); all other variables were treated as normally distributed. Significant variables appear in bold. ^†^ indicates deviance values for variables analyzed using binomial error structures.

Response Variable	Predictors	Mean Sq	DF	F Value	*p*-Value
Larval growth rate	Neonicotinoid treatment	6.30 × 10^−4^	4	1.24	0.294
	**Milkweed species**	**1.95 × 10^−3^**	**2**	**7.69**	**<0.001**
	Block	3.30 × 10^−4^	1	2.61	0.133
	Treatment:MWSpecies	2.53 × 10^−3^	6	1.99	0.680
Pupal deformity (0–3)	**Neonicotinoid treatment**	**1.51 × 10^+1^**	**4**	**39.59**	**<0.001**
	**Milkweed species**	**2.98**	**2**	**7.82**	**<0.001**
	Genetic lineage	5.25 × 10^−1^	4	1.38	0.242
	Block	2.58 × 10^−1^	1	0.68	0.411
	**Treatment:MWSpecies**	**4.55**	**6**	**11.95**	**<0.001**
Grip strength	Neonicotinoid treatment	4.83 × 10^−2^	4	2.29	0.062
	Milkweed species	8.38 × 10^−3^	2	0.4	0.673
	**Sex**	**3.65 × 10^−1^**	**1**	**17.27**	**<0.001**
	Block	2.23 × 10^−3^	1	0.11	0.770
	Treatment:MWSpecies	3.14 × 10^−2^	6	1.49	0.185
Wing area	**Neonicotinoid treatment**	**9.15 × 10^+3^**	**4**	**2.71**	**0.032**
	**Milkweed species**	**2.26 × 10^+4^**	**2**	**6.69**	**0.002**
	**Sex**	**2.22 × 10^+4^**	**1**	**6.57**	**0.011**
	Block	4.21 × 10^+3^	1	1.25	0.281
	Treatment:MWSpecies	3.99 × 10^+3^	6	1.18	0.318
Proportion eclosed	**Neonicotinoid treatment**	**NA**	**4**	**20.22 ^†^**	**<0.001**
	Milkweed species	NA	2	4.03 ^†^	1.335
	**Genetic lineage**	**NA**	**4**	**16.93 ^†^**	**0.002**
	Block	NA	1	0.39 ^†^	0.534
	**Treatment:MWSpecies**	**NA**	**6**	**14.05 ^†^**	**0.029**

## Data Availability

Data supporting these results are openly available in FigShare at [https://doi.org/10.6084/m9.figshare.16930189.v1; https://doi.org/10.6084/m9.figshare.16930147.v1] [75,76].

## Supplementary Materials

The following are available online at https://www.mdpi.com/article/10.3390/insects12110999/s1, Supplemental Methods for bee bioassay study; Table S1: Summary of prior studies of neonicotinoid effects on monarchs following exposure at the larval stage; Table S2: Experimental design for monarch larval neonicotinoid study; Table S3: Results from bumblebee exposure trial; Figure S1: Diagram of the flight mill apparatus; Figure S2: Photo of larval rearing design for the second experiment; Figure S3: photographs of normal and abnormal monarch pupae and pupation; Figure S4: Response of bumble bees to neonicotinoid exposure; and Figure S5: Time to pupation for experiment 2.
