# Supplementary material for: Host Plant Species Mediates Impact of Neonicotinoid Exposure to Monarch Butterflies
Source: Insects. 2021 Nov 6;12(11):999. doi: 10.3390/insects12110999 (PMC8623494; doi:10.3390/insects12110999)
Supplement: Supplementary file 1 [file insects-12-00999-s001.zip › insects-1359461-supplementary.pdf]

insects

MDPI

Supplementary Materials

1. Supplemental Methods: Bioassay in Wild Bees

1.1. Methods for Confirming Spiked Solution Toxicity Using Wild Caught Bumble Bee Bioassay

To confirm the toxicity of the neonicotinoid doses used here, wild caught *Bombus impatiens* were exposed to 0, 5, 50, and 500 ng/ml clothianidin and imidacloprid. Bees were captured while foraging at the UGA campus in Athens, GA USA, and were exposed within 1 hr. On average, the bumblebees used for this bioassay were of similar mass to monarch butterflies (Prouty, *unpubl. data*). We used the same stock solutions as for Experiment 2, to make a 20% (spiked) honey water solution with the specified neonicotinoid dose. The neonicotinoids were added directly into the already mixed 20% honey water solution. Bees were placed into clear acrylic containers (15×10×10cm) with mesh lids, to which we added sponges soaked in the honey water solution placed inside petri dish bottoms. We placed 4 bees into each container, with 1 container for each treatment. Containers were checked every 40–60 mins over a 4hr period to record the activity of each bee as: flying, active/crawling, standing, twitching (while lying on side), or dead (Table S3). Bees were frozen at 20 °C after observations were concluded.

1.2. Results for Confirming Spiked Solution Toxicity Using Wild Caught Bumble Bee Bioassay

After 4 hours, all four *B. impatiens* in the 0 ng/ml (control, honey water only) treatment remained actively flying, and had to be chilled at 14 °C prior to removal. For those exposed to imidacloprid 28 ng/ml applied dose, most bees remained active for the first 2 hrs. By 4 hours, the bees became inactive and showed signs of persistent twitching (Figure S4). At the 386 ng/ml imidacloprid applied dose, all bees became inactive or were twitching after 2 hours of exposure, and one bee died by the end of the 4 h interval. For those exposed to clothianidin, all bees from both the 36 and 531 ng/ml applied dose died before the end of 4 hrs. For the 36 ng/ml applied dose, all bees remained alive until 1.5 hours post-exposure, but for the 531 ng/ml applied dose, most bees died after just 1 h (Figure S4). This bioassay demonstrated the toxicity of the neonicotinoid solutions used in this study.

Table S1. Summary of prior studies of neonicotinoid effects on monarchs following exposure at the larval stage. Studies are identified by the type of neonicotinoid that was used and the milkweed species. If a study included multiple species of milkweed or neonicotinoids it was separated into multiple rows. LC50s are listed in the units each paper represented them in, with ppb = ng/g and ppm = μg/g. Exposure method indicates the way insecticides were applied to monarchs. Overall, all studies differed in terms of their exposure method, milkweed species used, and the stage at which monarchs were exposed, which led to differences in LD50.

Author	Neonic	Milkweed	Stage Exposed	LC50	Survival	Sublethal effects	Exposure Method
Pecenka and Lundgren 2015	Clothianidin	Swamp *A.incarnata*	1st, 2nd instar + 36 hr	9.8 ppb (ng/g)	NA	0.5 ppb +	solution on 1 cm diameter leaf discs
Krischik et al. 2015	Imidacloprid	Tropical *A.curassavica*	early instar until death (max 7 days)	NA	reduction at 8 ppm (μg/g) +	NA	Soil
Bargar et al. 2020	Clothianidin	Swamp *A.incarnata*	Newly hatched until death or pupation	47 to 205 ng/g	NA	177 ng/g +	Soil
Krishnan et al. 2020	Imidacloprid	Tropical *A.curassavica*	2nd, 3rd instar + 48 hrs	5.1,17 μg/g (2nd,3rd instar)	NA	0.75 μg/g (third ins.) +	Foliar

Krishnan et al. 2020	Clothianidin	Tropical *A.curassavica*	2nd, 3rd instar + 48 hrs	4.2,7.8 µg/g	NA	NA	Foliar
Krishnan et al. 2020	Thiamethoxam	Tropical *A.curassavica*	2nd, 3rd instar + 48 hrs	3.5,5.6 µg/g	NA	4.8 µg/g +	Foliar
Olay-Arenas et al. 2020	Clothianidin	Common *A.syriaca*	Newly hatched	N/A	Reduction at 56.55 ng/g	N/A	Foliar, leaf clippings
Wilcox et al. 2021	Clothianidin	Swamp *A.incarnata*	From eggs to pupation	NA	NA	None up to ~10 ppb recovered	Soil

Table S2. Experimental design for monarch larval neonicotinoid study. Each treatment group included 5 genetic lineages. Concentrations are ng of neonicotinoids/ml of spiking solution. Recovered concentrations on leaves are shown in Table 1.

**Milkweed**	**Swamp**					**Tropical**					**Common**		
**Treatment**	**Control**	**Clothianidin**		**Imidacloprid**		**Ctrl**	**CL**		**IM**		**Ctrl**	**CL**	**IM**
Conc(ppb)	0	50	500	50	500	0	50	500	50	500	0	500	500
# Monarchs	24	24	24	24	24	15	15	15	15	15	15	15	15

Table S3. Results from bumblebee exposure trial as described in the methods of the main text. Data were collected hourly in the form of general observations. Time indicates minutes after exposure to neonicotinoids. IM corresponds to imidacloprid, CL to clothianidin and numbers are the concentrations in ng/ml. See Table 1 for chemical analysis of spiking solutions. "Active" indicated that the bee was seen walking around the cage at a normal speed. Bees that were "twitching" were seen standing still with legs twitching or shaking. Observations were made approximately every 40–50 minutes.

**Time**	**Control**	**IM 28**	**IM 386**	**CL 36**	**CL 531**
10	4 active	4 active	4 active	4 active	2 dead, 2 twitching
50	4 active	4 active	4 active	3 active, 1 immobile	3 dead, 1 twitching
90	4 active, flying	4 active	4 immobile/twitching	1 dead, 3 active	4 dead
140	4 active	1 twitching, 3 active	2 twitching, 2 slowly crawling	3 dead, 1 crawling slowly	-
190	4 flying	2 twitching, 2 not moving	1 dead, 2 twitching, 1 standing	3 dead, 1 on sponge	-
250	4 flying	3 twitching, 1 moving	1 dead, 3 twitching on backs	4 dead	-

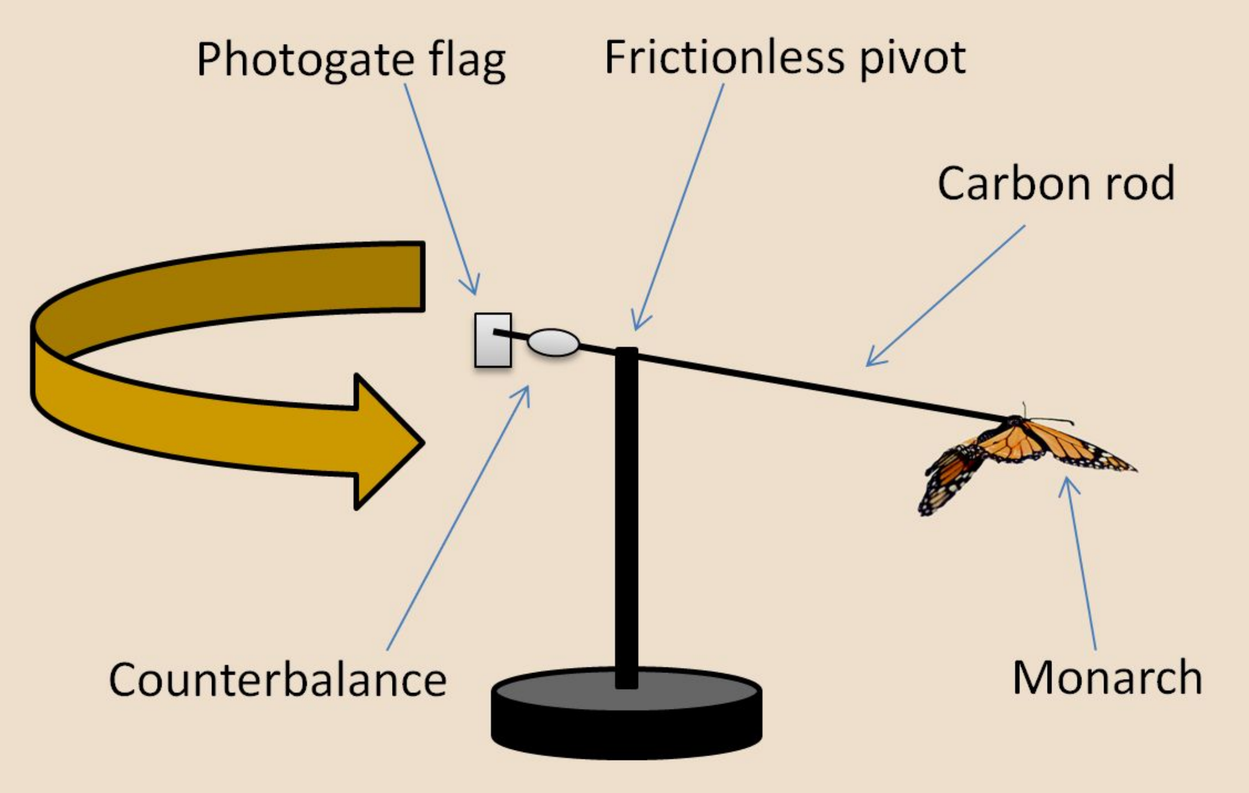

Figure S1. Diagram of the flight mill apparatus used to induce powered flight in experimental monarchs. Monarchs were attached to a 90 cm lightweight carbon rod with a diameter of 3 mm (4.23 m circumference flight path) balanced on a frictionless steel pivot, and with a moveable counterbalance to account for variation in each monarch's weight. We tethered monarchs to one end of the horizontal rod using a lightweight steel fishing line. A 5 cm flag at the opposite end of the rod passed through an infrared beam on a photo-gate to estimate flight time for each revolution, using CAPSTONE Software (Pasco). Credit: Andrew K. Davis.

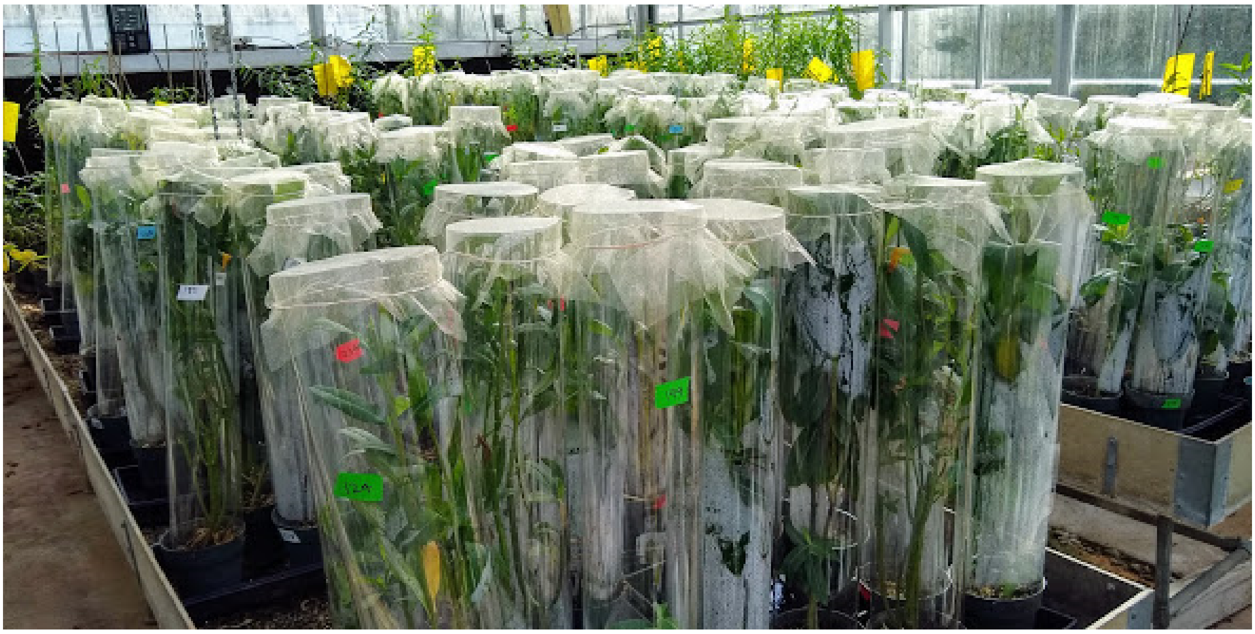

Figure S2. Photo of larval rearing design for the second experiment. In the image are plants covered by tubes and a mesh covering the opening at the top. Each tube contains one caterpillar. Tubes were roughly 1 meter tall, with a diameter of 15 cm, that were fit into the pots and covered with soil. Mesh screens were held in place with rubber bands.

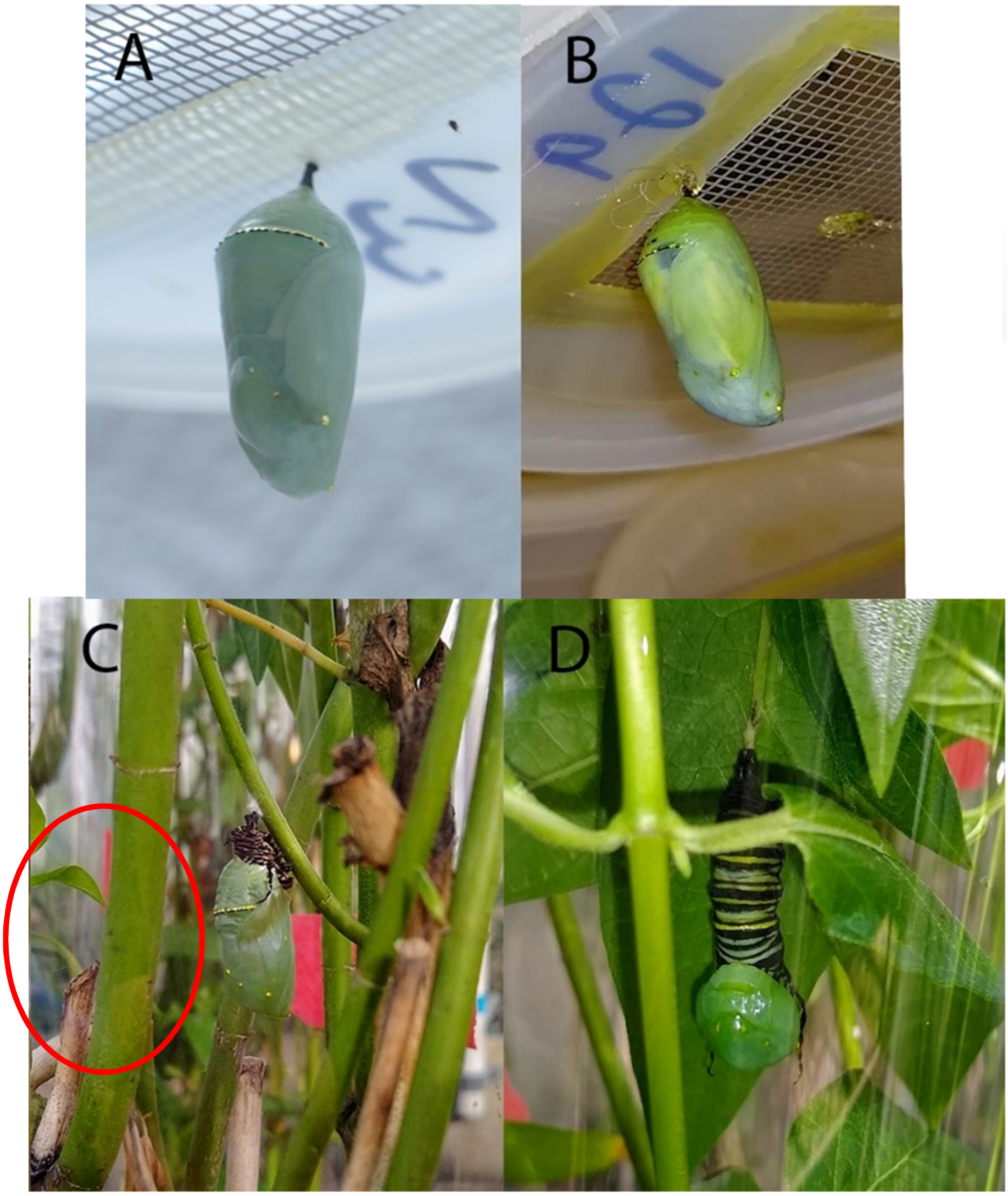

Figure S3. photographs of normal and abnormal monarch pupae and pupation. (**A**) Monarch pupa showing normal morphology with no sign of deformity. (**B**) Monarch pupa with slight discoloration and wrinkles (deformity score = 1). (**C**) Pupa showing substantial discoloration and wrinkles (deformity score = 2). (**D**) Pupa showing failed ecdysis (larval integuments remained on pupa as it hardened; deformity score = 3).

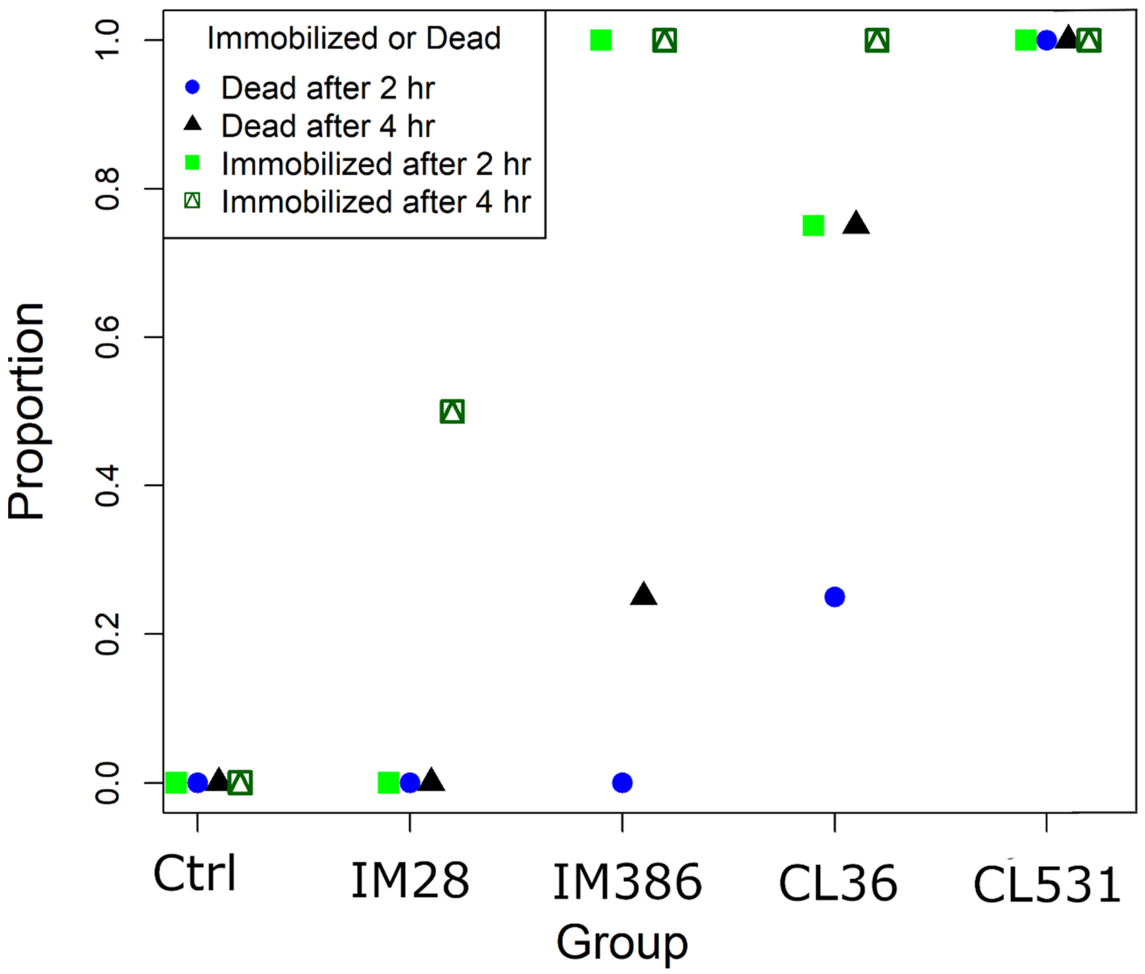

Figure S4. Response of bumble bees to neonicotinoid exposure. X-axis represents treatment groups, and each dot corresponds to the proportion of bumblebees that were either dead or immobile at each given time.

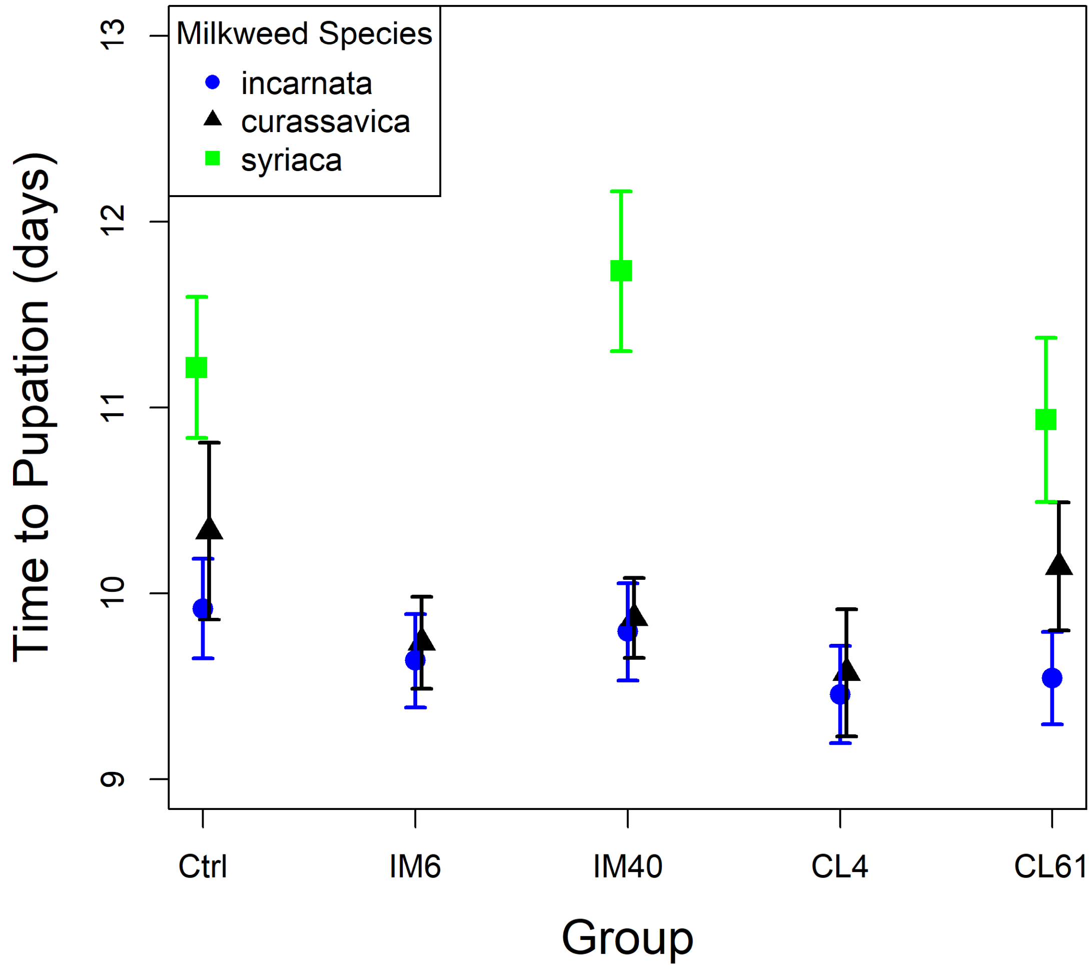

Figure S5. Time to pupation for experiment 2. The graph displays the duration from when monarchs were placed into enclosures until the date of pupation. IM corresponds to imidacloprid and CL to clothianidin. Numbers following the neonicotinoid indicate the recovered concentration on leaf tissues in ng/g, averaged between the samples of swamp and tropical milkweed, with common milkweed assumed to fall within the range. Swamp milkweed (*A. incarnata*) is in blue circles, tropical (*A. curassavica*) is in black triangles, and common milkweed (*A. syriaca*) is in green squares.
